# Adapting evidence-informed population health interventions for new contexts: a scoping review of current practice

**DOI:** 10.1186/s12961-020-00668-9

**Published:** 2021-02-05

**Authors:** A. Movsisyan, L. Arnold, L. Copeland, R. Evans, H. Littlecott, G. Moore, A. O’Cathain, L. Pfadenhauer, J. Segrott, E. Rehfuess

**Affiliations:** 1 Institute for Medical Information Processing, Biometry and Epidemiology, LMU Munich, Elisabeth-Winterhalter-Weg 6, 81377 Munich, Germany; 2 Pettenkofer School of Public Health, LMU Munich, Elisabeth-Winterhalter-Weg 6, 81377 Munich, Germany; 3 Centre for Development, Evaluation, Complexity and Implementation in Public Health Improvement (DECIPHer), School of Social Sciences, Cardiff University, 1-3 Museum Place, Cardiff, CF10 3BD Wales UK; 4 School of Health and Related Research (ScHARR), University of Sheffield, Regent Court, 30 Regent Street, Sheffield, S1 4DA UK; 5 Centre for Trials Research, Cardiff University, 4th floor Neuadd Meirionnydd, Heath Park, Cardiff, CF14 4YS Wales UK

**Keywords:** Adaptation, Complex interventions, Complexity, Systems thinking, Evidence-based, Evidence-informed, Implementation, Evaluation, Context, Population health

## Abstract

**Background:**

Implementing evidence-informed population health interventions in new contexts often requires adaptations. While the need to adapt interventions to better fit new contexts is recognised, uncertainties remain regarding why and when to adapt (or not), and how to assess the benefits (or not) of adaptation. The ADAPT Study aims to develop comprehensive guidance on adaptation. This scoping review informs guidance development by mapping and exploring how adaptation has been undertaken in practice, in public health and health services research.

**Methods:**

We searched seven databases from January 2000 and October 2018 to identify eligible studies for this scoping review and a related systematic review of adaptation guidance. We mapped the studies of adaptation by coding data from all eligible studies describing the methods, contexts, and interventions considered for adaptation. From this map, we selected a sample of studies for in-depth examination. Two reviewers extracted data independently into seven categories: description, key concepts, types, rationale, processes, evaluation methods, evaluation justification, and accounts of failures and successes.

**Results:**

We retrieved 6694 unique records. From 429 records screened at full text, we identified 298 eligible studies for mapping and selected 28 studies for in-depth examination. The majority of studies in our map focused on micro- (i.e., individual-) level interventions (84%), related to transferring an intervention to a new population group within the same country (62%) and did not report using guidance (73%). Studies covered a range of topic areas, including health behaviour (24%), mental health (19%), sexual health (16%), and parenting and family-centred interventions (15%). Our in-depth analysis showed that adaptation is seen to save costs and time relative to developing a new intervention, and to enhance contextual relevance and cultural compatibility. It commonly follows a structured process and involves stakeholders to help with decisions on what to adapt, when, and how.

**Conclusions:**

Adaptation has been undertaken on a range of health topics and largely in line with existing guidance. Significant gaps relate to adaptation of macro- (e.g., national-) level interventions, consideration of programme theories, mechanisms and contexts (i.e., a functional view of interventions), nuances around stakeholder involvement, and evaluation of the adapted interventions.

*Registration* Open Science Framework, 2019, osf.io/udzma.

## Background

Population health interventions include policies and programmes in public health and health services research that aim to change the population distribution of risk [1]. Implementation of evidence-informed population health interventions (i.e., those interventions that have already been assessed to be effective) in new contexts may save financial and human resources compared with the development of new interventions for each context. However, since these interventions are implemented in complex systems and thus shape and are being shaped by their context [2, 3], adaptations often need to be made to accommodate different contextual features, resources, and infrastructure.

Adaptation is commonly defined as intentional modification(s) of an intervention to achieve better fit with a new context [4, 5]. Different types of adaptations have been discussed in the literature, including adaptations to the content of the intervention, the way it is delivered, or the surrounding context [6]. Cultural adaptation specifically focuses on maintaining the cultural relevance of an intervention when delivering it to different population groups [7]. Inherent to the discussions of adaptation are several debates and uncertainties. First, adaptation is often presented to be in tension with the “fidelity” of the intervention (i.e., delivery as intended) [8]; the associated uncertainty is how and to what degree to adapt, so as not to compromise intervention effects [9]. A related debate is how best to define and operationalise fidelity, such as in relation to intervention form versus function; the former concerns the specific content and delivery of the intervention, and the latter its mechanisms and theoretical principles [10, 11]. Second, while the need to adapt an intervention to enhance its fit with the new context may seem intuitive, evidence on the successes (or not) of adaptation (i.e., whether adaptations increase the likelihood of the intervention working in the new context) is mixed. Some studies provide evidence in favour of adapted interventions [12, 13]; others suggest no added benefits associated with extensively adapted interventions [14]. This indicates that not all adaptations are warranted and that the extent of adaptations in a given context should be made carefully. It might also be that the intervention simply does not fit the new context even with careful adaptation, or that the original evidence of effects was flawed. This creates uncertainty regarding why and when to adapt (or not), and how to decide on the need for and the extent of adaptation, as well as when uncertainties regarding intervention-context fit are sufficient that a new full evaluation is warranted.

To inform such decisions, the ADAPT Study aims to develop comprehensive and consensus-based guidance on adaptation [15]. Drawing on best practices in guidance development [16], the study follows a phased approach, including literature reviews to identify current guidance as well as current practice (phase 1), qualitative interviews and expert consultations (phase 2), and consensus development methods (phase 3).

We first conducted a systematic review of existing guidance on adaptation and found 35 guidance papers published since 2000 [5]. Despite broad agreement on terminology, types, and steps of adaptation, our review revealed major gaps. Specifically, most of the papers did not consider substantial contextual changes, such as those associated with transferring interventions across countries and continents, lacked adequate theorisation of intervention mechanisms and contextual interactions in the replicability of effects, and failed to describe strategies for re-evaluating adapted interventions (e.g., feasibility study vs randomised trial). It is therefore important to also examine how adaptation is conducted in practice and how it compares with and complements the gaps in the existing guidance.

So far, there is little research looking into the real-world practice of adaptation, specifically how adaptations are justified, implemented, and evaluated in practice, and who is involved. A recent systematic review examines reported reasons, common steps, and outcome measures of adaptations in public health [17]. While it provides informative descriptive statistics on these aspects, the review does not explore in-depth the rationale and procedures of adaptation, nor does it draw on literature beyond public health. In this scoping review we will first develop a comprehensive map of existing primary studies on adaptation from across public health and health services research and then apply a thematic qualitative approach to explore in-depth the content of a selected sample of those studies. Specifically, we aim to examine in-depth (i) key concepts used in intervention adaptation, (ii) types of adaptations, (iii) the rationale for or against adaptation, (iv) procedures undertaken to adapt the intervention, including stakeholders involved in the process, and (v) approaches used to assess the effectiveness of an adapted intervention. We will then examine how the key aspects and procedures of adaptation as described in the selected cases from practice compare with the existing recommendations as synthesised in the systematic review of guidance on adaptation [5]. This will help draw pragmatic suggestions for further examination and agreement in the ADAPT Study.

## Methods

We have used the methodological framework for scoping reviews by Arksey and O’Malley [18], as further modified and enhanced by Levac and colleagues [19], to guide the review. Accordingly, our review followed these steps: (i) specifying the research question by clarifying and linking it with the overall objective of the scoping review, (ii) identifying relevant studies through transparent balancing of breadth and practicability, (iii) selecting studies for inclusion, (iv) charting the data through an iterative process of data extraction and refinement of the form, (v) collating, summarising, and reporting the results using qualitative thematic analysis, where appropriate, and comparison of the findings with the review aims, and finally, (vi) consulting with stakeholders as an optional step. This review is reported following the Preferred Reporting Items for Systematic Reviews and Meta-Analysis (PRISMA) extension for Scoping Reviews (see Additional file 1 for the completed PRISMA-ScR checklist) [20]. The review protocol was pre-registered on the Open Science Framework (osf.io/udzma).

### Eligibility criteria

We included studies that (i) were a primary study describing an adaptation process and/or an evaluation of an evidence-informed intervention adapted to a new context, (ii) focused on public health and/or health service interventions, (iii) were published from 2000 onwards, when the topic of evidence-informed interventions and complexity came to the fore, and (iv) were available in English, German, French, Italian, Spanish, Russian, or Swedish, as these languages could be comprehensively covered by the study team members. We excluded studies that (i) reported interventions that had been designed de novo for a specific context or population containing components used in other interventions and/or (ii) examined specific clinical procedures, such as surgery. Table 1 provides further clarifications of the eligibility criteria.

**Table 1:** Eligibility criteria

Criterion	Definition
Document type	Peer-reviewed research papers Non-peer-reviewed research documents (e.g., dissertations, theses, book chapters)
Document focus	Primary studies describing a process of adaptation of an evidence-informed intervention to a new context Primary studies evaluating an evidence-informed intervention adapted to a new context (e.g., process and/or outcome evaluation)
Adaptation	Modifications made to the content of interventions AND/OR Modifications made to the delivery of interventions AND/OR Modifications made to the context in which interventions are delivered
New context	Drawing on the Context and Implementation of Complex Interventions (CICI) framework dimensions [67], context is characterised by differences in geographical, epidemiological, socio-cultural, socio-economic, ethical, legal, and/or political determinants NB: Research papers which describe scale-up of interventions will be included only if the scale-up is described in relation to changes in any of the foregoing contextual features (e.g., taking interventions tested in a specific district for implementation in other districts, which differ in their contextual profile, such as population and socio-economic determinants)
Population health interventions	Interventions, programmes, and policies which seek to change the population distribution of risk/health outcomes These interventions can be delivered to whole populations or sub-groups defined based on specific characteristics (e.g., age, increased levels of risk) Interventions may encompass public health or health services research
Year	Research papers published from 2000 onwards
Language	Papers written in English, German, French, Italian, Russian, or Spanish
Geographical location	Any

### Search strategy

We drew on the searches from our related systematic review of adaptation guidance [5]. For that, we conducted searches of scientific databases and grey literature sources up to October 12, 2018: Applied Social Science Index & Abstracts (ASSIA), Conference Proceedings Citation Index—Social Science & Humanities (CPCI-SSH), Dissertations and Theses Global: The Humanities and Social Sciences Collection; EMBASE; MEDLINE and Epub Ahead of Print, In-Process & Other Non-Indexed Citations, Daily and Versions; PsycINFO; and Social Science Citation Index (SSCI). The broad search strategy was developed around search blocks focusing on (i) implementation, evaluation, and dissemination, as well as methods of evidence-informed interventions, and (ii) adaptation (see Additional file 2 for the search strategy).

### Screening and mapping of studies

**Fig. 1:**
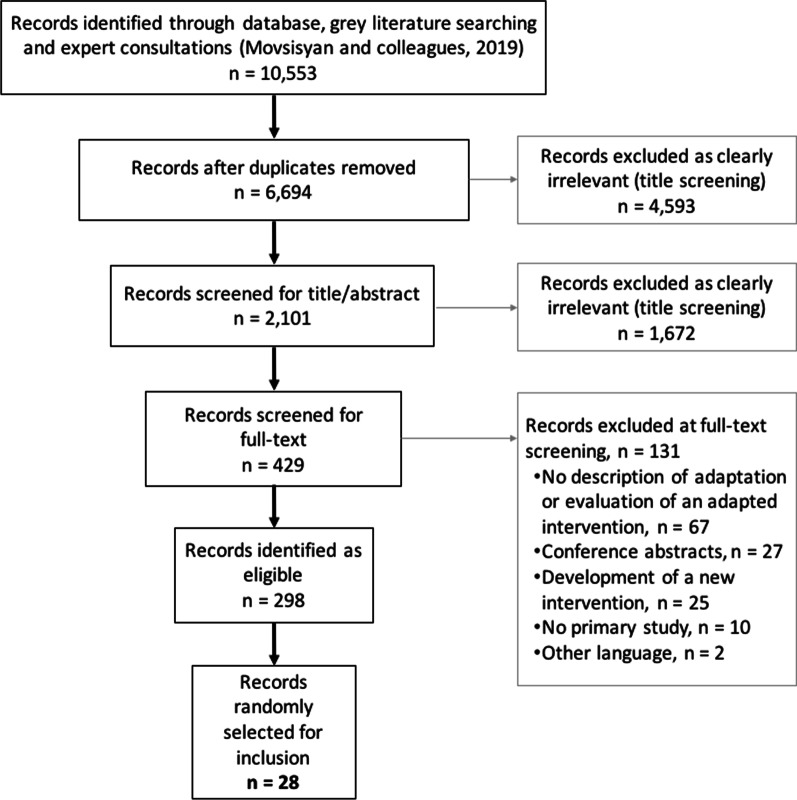
Flow chart of the identification and selection of studies

For the previous systematic review, we identified 6694 records (see Fig. 1 for the PRISMA flow diagram). One reviewer first screened the titles to remove clearly irrelevant studies. Subsequently, two reviewers independently screened 2101 titles and abstracts and identified potentially relevant guidance papers, as well as 429 studies reporting on the adaptation of specific interventions (with or without an associated evaluation). For this scoping review, one reviewer (shared among AM, LA, HL, and LC) screened the full text of these 429 records again in 2019 and, for eligible studies, coded them. For quality assurance, a 5% subset of studies was independently screened and coded by two reviewers. Throughout the process, uncertainties were noted, discussed, and resolved with recourse to the other members of the review team.

We developed a map describing the focus and scope of the 298 eligible studies. In doing so, we coded the following information: (i) authors and year of publication, (ii) methods (e.g., whether the study described an adaptation process or evaluated an adapted intervention in a new context or both), names of the (iii) original and (iv) adapted interventions, (v) intervention topic area (e.g., parenting and family-centred interventions, substance use), (vi) level of intervention (i.e., macro-, meso-, micro-level^1^), (vii) specified contextual change (e.g., transferring interventions from one country to another, such as from the United States to Sweden, or from one ethnic group to another within the same country), (viii) type of adaptation (e.g., content, delivery), and finally, (ix) use of existing adaptation guidance (where applicable) [5].

### Selection of studies for in-depth analysis

Given the large number of eligible studies, we selected a sample for more in-depth examination in this scoping review. To ensure diversity in the sample, we stratified the studies from the map based on (i) the methodological approach (i.e., adaptation process vs evaluation), (ii) the level of intervention (i.e., micro, meso, and macro), and (iii) the topic area (i.e., health behaviour interventions, substance use, parenting, mental health, sexual health, and others). We then randomly selected one study per topic area from each level of intervention and methodological approach.

### Data extraction for in-depth analysis

Guided by the review objectives, a data extraction form was developed and piloted by two independent reviewers (AM and LA) on two eligible studies (see Additional file 3). Uncertainties during piloting were noted and discussed with additional reviewers. Data from this sample of studies were extracted independently by two reviewers (AM and LA). Disagreements were resolved through discussion and consultation with a third reviewer when necessary. As per the guidance for conducting scoping reviews, we did not assess the methodological quality of the included studies [21].

We extracted descriptive information on the study, including the author, year, title, and publication source (already extracted as part of the mapping above) and information across seven categories: (1) key concepts and nomenclature used; (2) types of adaptation undertaken by the researchers; (3) reported justifications for the adaptations; (4) processes that researchers undertook to adapt the intervention, including the role of the intervention developers and other stakeholders, as well as the extent of using existing adaptation guidance; (5) methods employed by the researchers to evaluate the adapted interventions, (6) justifications for decisions regarding the extent of evaluation required in the new context, and finally, (7) narrative accounts for adaptation failures or successes.

### Data summary based on mapping and in-depth analysis

To summarise the data coded for the map, we used frequency analysis. This map can be considered a database of existing studies of adaptation.

To describe the data obtained through the in-depth analysis of selected studies, we used tabular and thematic qualitative approaches. Employing cross-case tabulation [22], we first sorted the data based on the pre-defined categories of the data extraction form (e.g., all data extracted for the “types of adaptation” were sorted together). We then charted the extracted data to examine how data in each category were described across the included studies (e.g., what types of adaptation were reported in different studies). To do so, we used an inductive analytic approach and coding (e.g., content adaptation as a type of adaptation) [23]. Using these, narratives were developed describing data in each category. These were developed by one reviewer (AM), reviewed by all team members, and revised based on their feedback.

Below we summarise the data extracted in categories 1–6. We did not find any discussion of adaptation failures or successes in the selected studies, and therefore omitted category 7. We describe data for categories 5 and 6 in the extraction form related to the methods of evaluation and justifications for choosing those methods under the combined category of *evaluation of an adapted intervention*. *Stakeholder involvement* emerged as a widely discussed topic, and we therefore added this as a new category.

### Stakeholder consultation

This scoping review is conducted as part of the ADAPT Study—a multi-phase project to develop overarching guidance on adaptation. The findings from this review will be further examined through qualitative interviews and an international Delphi panel with key stakeholders, including researchers, practitioners, policymakers, funders, and journal editors. The guidance will then be finalised based on several rounds of consultations and revisions [15].

## Results

### Map of adaptation studies

**Fig. 2:**
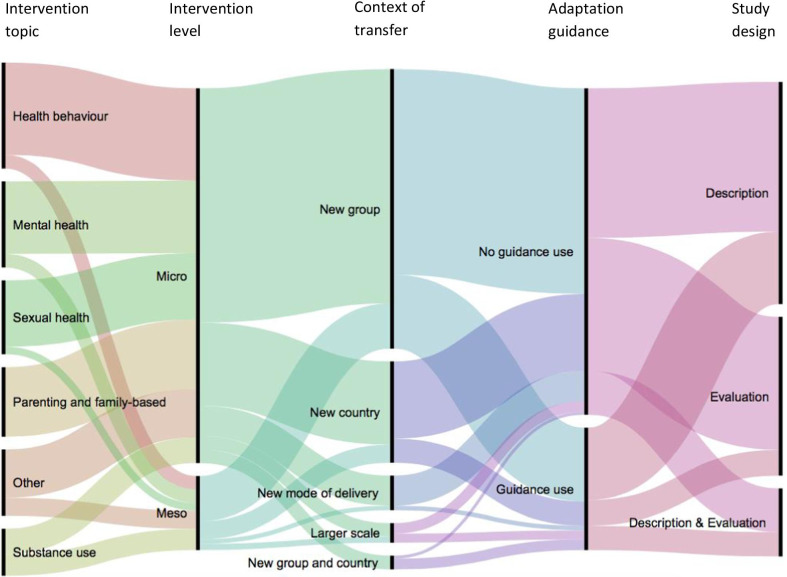
Map of adaptation studies (*n* = 298). This alluvial plot shows how data are distributed within and across the categories of the map. For example, it shows that health behaviours (darker pink) represent one of the intervention topics and that most of the studies with this topic reported micro-level interventions. Similarly, we can see that most studies adapting an intervention to a new country (dark purple) do not report using any adaptation guidance

We identified 298 eligible studies for the map (see Fig. 2). Table 2 shows their characteristics according to the level of intervention. Additional file 4 provides further details regarding the map, including the topic area and focus of the intervention, adaptation context, and types and framework considered in each study. Most studies targeted micro-level interventions (84%), provided a description of adaptation (50%), did not report using adaptation guidance (73%), and described a transfer of an intervention to a new target group within the same country (63%) (most of these were within the United States). Studies covered a range of topic areas, including health behaviour interventions (23.5%), mental health (19%), sexual health (16%), parenting and family-centred interventions (15%), and substance use (10%).

**Table 2:** Descriptive analysis of the studies included in the map

	Micro (*n* = 249)	Meso/Macro^a^ (*n* = 49)	Total (*n* = 298)
Intervention topic area and focus, *n* (%)			
Health behaviour (risk and protective)	61 (25)	9 (18)	70
Substance use	17 (7)	14 (29)	31
Parenting and family-centred	46 (19)	0 (0)	46
Mental health	48 (19)	9 (18)	57
Sexual health	44 (18)	5 (10)	49
Others	33 (13)	12 (25)	45
Study design, *n* (%)			
Description	130 (52)	18 (37)	148
Evaluation	85 (34)	20 (41)	105
Description and evaluation	34 (14)	11 (22)	45
Context of intervention transfer, *n* (%)			
Transfer to a different target group	156 (63)	30 (61)	186
Transfer to a new country	55 (22)	12 (25)	67
Transfer to a new country and a new target group	9 (4)	0 (0)	9
Change in the mode of delivery	20 (8)	3 (6)	23
Larger-scale implementation	9 (4)	4 (8)	13
Use of adaptation guidance, *n* (%)			
Yes	70 (28)	11 (22)	81
No	179 (72)	38 (78)	217

We found only one paper targeting a macro-level intervention and therefore report this together with meso-level interventions

### Characteristics of selected studies

Overall, 28 studies were selected for in-depth analysis (see Table 3). Since we found only one study describing a macro-level intervention (see Table 2) and no study describing a meso-level intervention, our sampling within these categories yielded 23 studies. To enlarge our sample, we randomly selected an additional five studies from those describing the transfer of an intervention to a new country, because one of the gaps in the existing guidance papers on adaptation was that they do not sufficiently address adaptation between countries [5].

**Table 3:** Characteristics of selected studies (*n* = 28)

First author, year	Original intervention name	Adapted intervention name	Study method	Topic	Level of intervention	Context		Adaptation guidance used	Theoretical principles of the adaptation approach
						Original	Adapted		
Audit 2017 [36]	Adherence Support Workers programme	NR	Description and pilot-testing	HIV	Micro	People living with HIV (PLHIV) in Malawi	PLHIV in rural Mozambique	Wingood et al. 2008 (ADAPT-ITT) [61]	sIMB-CIM
Barrett 2015 [42]	Preventure programme	Preventure programme	Description	Substance use	Micro	High-risk youth in UK	High-risk Australian adolescents	NR	NR
Benjamins 2010 [24]	Coordinated School Health model	NR	Description and evaluation: pre–post-test	Nutrition and physical activity (obesity)	Meso	US public schools	Jewish schools in Chicago	NR	CBPR
Benson-Florez 2017 [47]	Behavioral Activation Latino	Behavioral Activation Latino (for families)	Description and evaluation: family case study	Mental health: depression and family well-being	Micro	Latino individuals	Latino families	Bernal and Domenech Rodriguez 2012 [62]	Ecological validity model
Betts 2018 [25]	Diabetes Prevention Program Group Lifestyle Balance (DPP GLB)	Group Lifestyle Balance program Adapted for individuals with Impaired Mobility (GLB-AIM)	Description	Nutrition and physical activity (diabetes)	Micro	General US population	People with impaired mobility in the USA	NR	CBPR
Crooks 2018 [37]	Mental Health First Aid Basic	Mental Health First Aid First Nations	Evaluation: pre–post-test	Mental health crisis	Meso	Australian general population	First Nations peoples in Canada	NR	CBPR
Evans 2015 [38]	NR	RITCh Study Tobacco Dependence Treatment Manual and Toolkit	Description and preliminary evaluation: pilot study	Tobacco dependence	Micro	General US population	Lower socio-economic status African American people	Barrera and Castro 2006 [63]	CBPR
Garbers 2016 [44]	Get Yourself Tested	Get Yourself Tested	Description and evaluation: pre-experimental	STD	Meso	General US population	Black and Latino sexual minority youth in the USA	NR	NR
Goode 2012 [26]	Logan Healthy Living Program (LHLP)	Optimal Health Program (OHP)	Descriptive case study	Nutrition and physical activity (diabetes/hypertension)	Micro	Research setting	Practice setting in Australia	NR	Roger’s diffusion of innovations theory
Grau 2013 [43]	Phaphama	NR	Description and pilot evaluation	HIV/STI	Micro	US and South African population	Russian STI clinic patients	NR	NR
Highfield 2015 [39]	Mobile mammography programme	Mobile mammography programme	Description and evaluation: quasi-experiment	Cancer screening: Mammography	Micro	General US population	African American women	Bartholomew et al. 2016 (Intervention mapping) [64]	NR
Horn 2008 [27]	Not On Tobacco program (N-O-T)	American Indian Not On Tobacco program (AI NOT)	Description	Tobacco dependence	Meso	General US population	North Carolina American Indians	NR	CBPR
Inam 2015 [33]	Promoting Alternative Thinking Strategies (PATHS)	Promoting Alternative Thinking Strategies (PATHS)	Description and pilot evaluation	Mental health	Micro	General US population of children	Pakistani children in Islamabad	NR	NR
Kim 2013 [28]	Dietary Approaches to Stop Hypertension (DASH)	Dietary Approaches to Stop Hypertension for Koreans (K-DASH)	Evaluation: pre–post-test	Nutrition/diet (hypertension)	Micro	General US population	US Korean immigrants (Korean Americans)	NR	CBPR
Kirk 2018 [45]	Casarett EBI	NR	Description and pilot evaluation	Hospice care referrals	Micro	Intervention delivered in nursing homes	Intervention delivered in community-based settings in the USA	Lee et al. 2008 (Planned Adaptation Model) [65]	NR
Kwong 2017 [49]	Breathmobile program/PADMAP	NR	Evaluation: pre–post-test	Asthma management	Meso	Large-scale clinic system	Small-scale clinic system	NR	NR
Macridis 2016 [29]	School Travel Planning (STP) process	NR	Description	Physical activity	Meso	General schools in Canada	Indigenous community in Kahnawake, Canada	NR	CBPR
Martinez 2014 [30]	HoMBReS (Men Maintaining Well-being and Healthy Relationships)	NR	Description	AIDS	Meso	Latino men living in rural North Carolina	Latino men living in Indianapolis, Indiana (urban setting)	McKleroy et al. 2006 (Map of Adaptation Process) [66]	NR
Matarazzo 2014 [34]	Window to Hope (WtoH)	NR	Description and pilot evaluation	Mental health	Micro	Australian civilians	US veterans with severe traumatic brain injury	NR	NR
Mejdoubi 2011 [35]	Nurse Family Partnership	VoorZorg	Description and evaluation protocol	Parenting	Micro	High-risk pregnant women in the USA	High-risk pregnant women in the Netherlands	NR	NR
Mellins 2014 [31]	Collaborative HIV Prevention and Adolescent Mental Health Program+ (CHAMP+)	VUKA (“Wake up” in isiZulu)	Description	HIV	Micro	HIV-infected youth in New York, USA	HIV-infected pre- and early adolescents in South Africa	NR	CBPR
Reddy 2017 [48]	Dangerous Decibels programme	Dangerous Decibels: Industry (DDI) programme	Description and evaluation: pre–post-test	Hearing conservation	Meso	Students at schools in New Zealand	Industry workers in New Zealand	NR	Ecological approach
Stevenson 2008 [51]	NR	NR	Evaluation: pre–post-test and cost-effectiveness	Road traffic injury	Macro	High-income countries	Guangzhou city, China	NR	NR
Tsey 2005 [50]	Family well-being	School-based family well-being	Description and evaluation: qualitative	Mental health	Meso	General adult population of Australia	Indigenous school children in remote communities in Australia	NR	Participatory action research
Turhan 2017 [46]	Healthy School and Drugs (HSD)	HSD programme adapted for special education (HSD-SE)	Evaluation: quasi-experimental study	Substance use	Meso	Regular schools in the Netherlands	Special schools in the Netherlands	NR	NR
Vandenhoud 2010 [52] (Poulsen 2010 [41])	Parents Matter! Program (PMP)	Families Matter! Program (FMP)	Evaluation	Parenting	Micro	Parents and their preteens in the USA	Parents and their preteens in rural Kenya	NR	NR
Venner 2016 [40]	Cognitive Behavioural Therapy, Motivational Interviewing, Community Reinforcement Approach	MICRA (Motivational Interviewing and Community Reinforcement Approach)	Pilot evaluation	Substance use	Micro	General non-Hispanic White populations in the USA	American Indian/Alaska Native (AI/AN) in the USA	NR	CBPR
Williams 2013 [32]	ATHENA (Adherence Through Home Education and Nursing Assessment)	NR	Description and pilot evaluation	HIV/AIDS	Micro	HIV patients in North America	HIV patients in Hunan Province in South Central China	NR	NR

*ADAPT-ITT* Assessment–Decision–Administration–Production–Topical Experts–Integration–Training–Testing, *CBPR* community-based participatory research, *EBI* evidence-based intervention, *HIV* human immunodeficiency virus, *NR* not reported, *sIMB-CIM* situated-Information Motivation Behavioural Skills Model of Care Initiation and Maintenance, *STD* sexually transmitted disease, *STI* sexually transmitted infection

The selected studies focused on a range of topics: mental health and parenting (*n* = 7), sexually transmitted diseases (*n* = 6), tobacco and substance use (*n* = 5), and nutrition and physical activity (*n* = 5). Seventeen studies were concerned with micro-level interventions, ten with meso-level interventions, and one study with a macro-level intervention. Seven studies provided a description of adaptation, seven evaluated an adapted intervention, and 14 provided both a description and an evaluation. Only six studies reported using a guidance paper as identified in our related systematic review on adaptation guidance. In terms of the contextual change, 12 studies described transferring an intervention into a new country, including both high- and low-and middle-income settings (e.g., transferring an intervention from UK to Australia, from the USA to Pakistan, or from Malawi to Mozambique), and 16 studies described adaptations across different population groups within the same country (e.g., transferring an intervention from general US population to African Americans with lower socio-economic status). The theoretical principles underpinning the adaptation were not often discussed: when reported, these included community-based participatory research (CBPR; *n* = 9), ecological approaches (*n* = 2), participatory action research (PAR; *n* = 1), Roger’s diffusion of innovation theory (*n* = 1), and the situated-Information Motivation Behavioural Skills Model of Care Initiation and Maintenance (sIMB-CIM; *n* = 1).

**Fig. 3:**
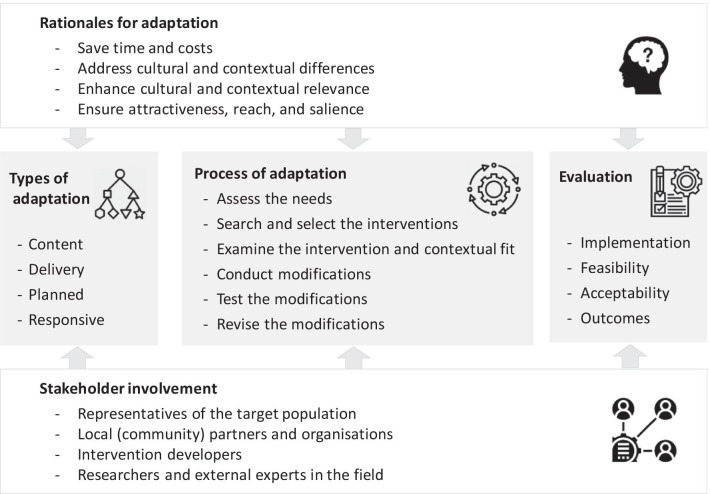
Key themes of adaptation in current practice

The key themes identified in the selected studies are presented in Fig. 3 and discussed below. Examples are summarised in Table 4.

**Table 4:** Examples from selected studies

Category	Examples
Types of adaptation: content modifications in response to profound cultural values and traditions	In their cultural adaptation of a mental health literacy intervention to the First Nations context, Crooks and colleagues describe modifications to foster community resilience by building upon specific healing resources of First Nations cultures [37] In an adaptation of an evidence-informed nursing intervention to improve medication adherence among people with HIV/AIDS in China, Williams and colleagues included family members in intervention activities to acknowledge the social importance of the family in China [32]
Types of adaptation: modifications made to the outward design	When transferring the intervention to prevent substance use and associated harms from UK to Australian adolescents, Barrett and colleagues changed the places (e.g., train station) and activities (e.g., athletics) on student leaflets to present more culturally appropriate situations [42]
Types of adaptation: modifications to how interventions are delivered	In their adaptation of HIV care and treatment in rural Mozambique, Audet and colleagues selected traditional healers as support workers to deliver the intervention When culturally adapting an intervention to reduce sexual risk behaviours among patients attending a STI clinic in St. Petersburg, Russia, Grau and colleagues used gradual and indirect introduction of role plays, as these exercises were not very common in the Russian context [36]
Process of adaptation: involving stakeholders to inform key decisions on modifications	When transferring a psychological intervention to reduce hopelessness after traumatic brain injury (TBI) from the Australian civilian context to that of US Veterans, Matarazzo and colleagues report organising a day-long stakeholder conference to learn about the intervention and reach consensus regarding necessary modifications. Stakeholders included the developer of the original intervention, professionals familiar with Veterans, rehabilitation or TBI, the Veterans Integrated Service Network, research staff, clinical psychologists, social workers, peer support specialists, and key community stakeholders [34]

#### Stakeholder involvement in adaptation

As shown in Table 3, a third of the sample of the studies explicitly described their adaptation process as grounded in the principles of CBPR and PAR. These highlight a partnership approach to research with equitable involvement of community, members of implementing organisations, and researchers in all phases of the research, as well as shared decision-making and joint ownership. Our analysis of the textual data also showed that involvement of different stakeholders in the adaptation process, particularly the local community, was the most frequently reported “strength” of the adaptation process [24–35]. Specifically, community engagement was viewed to facilitate the acceptability and compliance with the intervention in the new context, enhance responsiveness to local needs, and increase the likelihood of a sustained programme through empowerment and supporting local capacity and self-sufficiency. As Horn and colleagues reflect in their study describing adaptation of an intervention to reduce tobacco dependence among American Indians:

*“CBPR principles fostered sound research and meaningful results among a population historically exploited by research. Beyond the project’s quantitative data, the effort resulted in the development of new and successful partnerships, tobacco-addiction intervention programs (e.g., AI N-O-T), tools and resources tailored to community needs, and a multi-tribal interest in educating the youth and communities about tobacco addiction. The community also gained capacity to address the identified problem with greater self-sufficiency via increased grant-writing skills, evaluation knowledge, tobacco education, and financial resources”* [27].

Studies considered different stakeholder groups as important to involve throughout the adaptation process. These included ***representatives of the target population****, ****local (community) partners and organisations*** (such as those who would be delivering the intervention in a specific context) [24, 26–31, 36–41], ***practitioners*** [28, 30–33, 36, 39, 41–43] (such as clinicians, health professionals, psychologists), ***intervention developers*** [28, 30, 32–35], and ***researchers and external experts in the field*** [28, 30, 32–35]. Involvement of these different stakeholders was emphasised to a greater or lesser extent in different phases of the adaptation process. For example, representatives of the target group and local community partners were frequently emphasised when exploring the needs of the local community and the unique contextual features, as well as piloting of the adapted intervention and implementation. This ensured that interventions were modified with systematic consideration of key values and experiences. In contrast, the roles of intervention developers and experts were highlighted in providing information about the intervention, its components and theory, and in decisions regarding the specific modifications. This ensured that any changes made did not interfere with what the authors described as the intervention core components. Studies discussed different research methods and activities to involve stakeholders, including interviews, focus groups, surveys, theatre presentations, face-to-face meetings, and conferences.

A few studies also described **advisory boards** which comprised representatives of these different stakeholder groups overseeing and giving advice during all phases of adaptation [25, 29, 32, 34, 37, 39, 41, 43, 44]. For example, in the study on adaptation of a health behaviour intervention for people with impaired mobility, the 13-member national advisory board consisted of health professionals including rehabilitation physicians and occupational therapists, disability researchers specialising in weight loss, experts in human and organisational development, and representatives of community-based disability organisations [25]. The board oversaw and participated in making planned content modifications to the intervention, and provided ongoing advice on how to address the emergent issues identified during delivery.

#### Key adaptation terms and concepts used in practice

All studies used ***adaptation*** as the main term to denote changes made during intervention transfer to a new context, even though we had used several alternative terms in our search strategy. While studies did not provide definitions for adaptation, in a few instances adaptation was linked with the concept of evidence translation [26, 28, 35]. As noted by one of the studies, ***translation*** is a broader term which encompasses processes related to how evidence-informed interventions are adopted and adapted for use within community settings and systems [26]. From this perspective, translation was seen to sit within the broader ***dissemination*** umbrella, which also includes ***implementation***, ***evaluation***, and ***maintenance*** [26]. ***Tailoring*** was sometimes used interchangeably with adaptation. We did not find a study providing a specific definition for this term. ***Fidelity*** was another frequently used concept [25, 30–32, 34, 36, 39–41, 45, 46], and commonly referred to the extent of adherence to the original intervention protocol [32, 34, 39, 45] and its core components (i.e., essential components that make the intervention effective) [30, 31, 41]. Definitions for other concepts were not reported in the studies. For example, the term ***context*** was not defined but was broadly used to denote different circumstances, such as a geographical location (e.g., Australia vs sub-Saharan Africa), a cultural setting (e.g., First Nations context), or the geographical scope of an intervention (e.g., local vs global).

#### Presented rationales for adaptation

Rationales for adaptation were reported in 20 of the 28 studies. In most cases the description was brief and non-specific. In comparison to developing a new intervention in a specific context, adaptation of evidence-informed interventions was perceived ***to save costs and time*** related to de novo intervention development. Studies most frequently discussed the aims of adaptation to **enhance cultural and contextual relevance** [41]. Cultural and contextual insensitivity of an intervention was described as potentially contributing to null or negative intervention impacts and lack of acceptance and adherence to the intervention [37, 43]. In contrast, taking this into account could affect engagement with the intervention, its acceptability, feasibility, and outcomes [24–28, 31–43, 47]. As summarised in one of the studies,

*“Considering interventions are most successful when they are both based on science and culturally relevant (Castro et al., 2004), and considering the resources needed to develop and evaluate a new intervention, neither the exact replication of existing EBIs [evidence-based interventions] nor the development of new, culturally sensitive EBIs offers a sustainable solution. In light of this tension, the systematic, cultural adaptation of EBIs for new target populations and settings presents a way forward”* [41].

To ensure cultural relevance and contextual compatibility, studies highlighted the need to consider specific cultural values, beliefs, languages, and traditions (e.g., when adapting an intervention addressing tobacco dependence for Native Americans, for whom tobacco has a special role in spiritual and ceremonial events) [27, 33, 34, 36, 40, 43, 47], as well as unique structural characteristics of a new context, such as organisational capacity, and functional and environmental needs (e.g., when adapting an intervention addressing nutrition and physical activity for Jewish schools with specific dietary, behavioural, and belief systems) [25, 26, 39]. None of the studies provided explicit reflections on how relevant these cultural and structural factors are for intervention mechanisms and how they may interact with intervention mechanisms to affect implementation and outcomes in a new context. Other, less commonly mentioned reasons for adaptation included the need ***to ensure intervention attractiveness*** [40], ***reach*** [45], and ***salience*** [38].

#### Types of adaptation

***Content modifications*** were the most frequently described type of adaptation in the studies [24–34, 36–43, 45, 47, 48]. This often involved additions, deletions, or modifications of intervention components, such as specific activities and their duration. For example, in the adaptation of a health behaviour intervention designed for a general population to serve people with impaired mobility, Betts and colleagues changed all intervention sessions by revising the content, language, and delivery to make them “disability-friendly”, added a specific session on adaptive cooking, and revised the content of physical activity to explicitly address accessibility issues, such as through inclusion of tailored home-based activities [25]. Studies justified some of these content modifications as a response to profound social values and cultural traditions [27, 32, 37, 40]. Table 4 provides further examples. None of the studies reported on programme theories and how these may be modified during adaptation.

Studies also referred to the modifications made to the ***language and wording*** of the intervention [25, 28, 33, 34, 36, 40–43, 47] and its ***outward design***, such as when interventions involved the use of specific materials [24, 25, 27, 28, 33, 34, 37, 40–44]. Modifications to how interventions are ***delivered*** constitute another frequently discussed type of adaptation, including changes to the format of delivery and deliverers [25–27, 31, 36, 41, 43, 44, 49, 50] (see Table 4).

It should be noted that studies did not distinguish between adaptations that were initially planned and those that were actually undertaken, or offer any explanation for a possible discrepancy between these. We found only one study which differentiated between ***planned*** and ***responsive*** adaptations [25]. While planned adaptations included modifications that were agreed upon by the adaptation advisory board prior to intervention implementation (pre-intervention), responsive adaptations included unplanned modifications by the study team in response to emergent issues during the course of intervention delivery (concurrent with implementation). For example, during adaptation of the intervention for people with impaired mobility, additional conference calls were offered in the intervention group to complement the in-person sessions in response to the transportation barriers and declining attendance at the in-person sessions [25].

#### Process of adaptation

Only six studies reported using existing guidance to inform their adaptation process (see Table 3). Nonetheless, most of the studies describing intervention adaptation (and not an evaluation) reported a structured process consisting of **sequential phases and steps or key principles** [27, 28, 30–36, 38, 39, 41, 43, 45, 48]. Four studies that did not report a phased process, still described well-demarcated procedures [24–26, 44]. For example, when adapting an evidence-informed intervention to increase sexually transmitted disease (STD) testing among Black and Latino sexual-minority youth, Garbers and colleagues described procedures of formative research (including focus groups with the target group to identify their needs), followed by adaptation of the intervention materials, local implementation, and process and outcome evaluation [44]. Studies varied widely in the number of steps described (ranging from 2 to 20), in the level of detail provided, and in how they assigned specific procedures of adaptation across these steps.

Prior to undertaking modifications, many studies described some preparatory procedures. These commonly included ***assessment of the needs of the new context*** (e.g., through targeted literature reviews, stakeholder elicitation interviews, or focus groups) [27, 28, 30–32, 38, 39, 41, 43, 44, 48], ***searches for and selection of appropriate evidence-informed interventions ***[27, 30, 36, 39, 41, 48], and ***examination of the intervention, its core components, and contextual fit and misfit ***[24, 27, 28, 30, 32, 33, 39, 41, 43, 45]. Studies reported various ad hoc procedures for selecting appropriate evidence-informed interventions. These included ranking interventions based on specific criteria, such as reporting of a theory-driven approach, use of specific components, and prior implementation of the intervention in the region [36], and judgements of the fit of the intervention and its theory of change with key behavioural and environmental determinants, cultural features, and implementation resources of the new context [39]. Recommendations received through consultations with experts and/or networks of partners [41] was also one of the reported reasons for selecting an intervention for adaptation, as well as its inclusion in a registry of evidence-informed interventions, such as the Centers for Disease Control (CDC) Compendium of Evidence-Based HIV Behavioural Prevention Interventions [30]. None of the studies provided details on the types and nature of evidence that the original intervention had to have in order to be selected for adaptation.

For the specific steps or procedures of adaptation, studies most frequently reported ***conducting modifications to the intervention content or delivery*** [24–28, 31–36, 38, 39, 41, 43–45, 48], followed by ***preliminary testing of these modifications***, such as in a feasibility study [27, 28, 30, 32–36, 38, 39, 41, 43, 45], and making ***further revisions based on the testing*** [24, 25, 30, 33, 34, 38, 41, 43, 44, 48]. Decisions on the specific modifications were frequently reported to happen in consultation with expert or community advisory panels comprising a range of stakeholders (see Table 4).

#### Evaluation of adapted interventions

Studies did not report using any guidance to inform the evaluation of an adapted intervention.

Selected studies aiming to describe an intervention adaptation rather than its evaluation (*n* = 7, see Table 3) also described evaluation approaches. They reported process evaluations to inform intervention adaptations [30, 42], or pilot studies to examine the acceptability [31], feasibility of implementation [25, 31], and preliminary effectiveness of the adapted intervention [25, 27, 31]. A few of these, however, referred to a parallel ongoing larger efficacy/effectiveness study to examine the effects of the adapted intervention in the new context [25, 31], or recommended conducting such a study as the next step of evaluation [42].

Selected studies aiming to evaluate an adapted intervention reported conducting pilot, process, and outcome evaluations [24, 28, 32–40, 43–52]. Pilot evaluations were often seen as the first important step in testing the adapted intervention for feasibility (related to implementation, recruitment, and outcome measures), acceptability, and preliminary effectiveness (due to small samples). Process evaluations were reported to provide insights regarding the delivery and implementation of the adapted intervention and fidelity.

The subset of studies conducting an outcome evaluation reported using different designs, such as quasi-experimental and pre-test and post-test comparison designs. None of the studies applied a randomised controlled trial design; however, most studies referred to an associated efficacy/effectiveness trial either to be underway [34, 36, 38, 40, 43] or as a recommended or planned next step [28, 32, 33, 35, 45, 48]. While most studies did not provide explicit justification for the effectiveness trial, one paper explicitly noted the need for an effectiveness evaluation of an adapted intervention in light of the conducted adaptations potentially harming the effective components of the original intervention.

*“Best practice is to always evaluate an EBI used in a new setting, however, particularly one that has been adapted. Evaluation of adapted EBIs is recommended, since adaptation may harm the effective elements of an EBI (i.e., core elements). Besides this need for impact evaluation, there is a need to evaluate the feasibility and fidelity of intervention implementation in the new population and setting.”* [39].

Hybrid study designs, which simultaneously examine effectiveness outcomes and the implementation process (a term used in North America), was highlighted as an ideal design for the evaluation of an adapted intervention [39, 45]:

*“Our pilot study provides the initial evidence for conducting larger studies that would assess the effectiveness, impact, and sustainability of our adapted intervention … A hybrid trial, which investigates both intervention and implementation effectiveness, would be an ideal study design for subsequent research as it would allow for the simultaneous, systematic exploration of both intervention and implementation outcomes”* [45].

## Discussion

### Summary of main findings

This scoping review provides a map of 298 real-world studies of adaptation and examines in-depth the content of 28 studies describing and/or evaluating an intervention adaptation.

The map serves as a large database of adaptation cases describing the context of the adaptations, methods used, and intervention topic areas and levels considered. We found that the majority of adaptation studies have so far focused on micro-level interventions and have been implemented in the context of transferring an intervention to a new target group within the same country. Most adaptations do not report using formal guidance; a range of intervention topic areas have been considered for adaptation, including health behaviour, mental health, sexual health, parenting, and substance use.

Our in-depth analysis showed that adapting an intervention to a new context is seen in practice to save costs and time, compared with developing new interventions, and to enhance contextual relevance and cultural compatibility, compared with replicating interventions without adaptation. Adaptation is commonly reported as a structured process comprising different procedures largely grounded in the principles of CBPR and PAR, especially when cultural relevance is a key rationale for adaptation. Stakeholder involvement and local empowerment and ownership are therefore central to the practice of intervention adaptation. We found that most common procedures of adaptation in practice include needs assessment, intervention selection, and identification of the areas of fit and misfit (and thus areas requiring adaptation), followed by implementation of modifications to the intervention content, language or delivery, pilot testing, and informed revisions. Adapted interventions were commonly evaluated for implementation, feasibility, and outcomes; while we did not find any paper employing a randomised trial design for outcome evaluation, linked effectiveness trials were commonly reported to be ongoing or as a recommendation for future research.

### Current practice versus existing guidance in the context of the broader literature

Adaptation in practice seems to largely agree with the existing guidance and recommendations on adaptation [5]. As in the guidance papers, adaptation is seen as an efficient approach over de novo development of interventions for each specific context. In practice, adaptation is described as modifications to the intervention content and delivery. We did not find reporting of adaptations to context in the selected studies of adaptation, which was a type of adaptation described in the guidance papers. However, it was rarely discussed in the guidance papers and involved modifications to the elements of the broader system in which interventions are implemented (e.g., changes to funding and contracting to support implementation) [53]. This may be indicative of a narrow perspective on context in the sample of studies focusing on micro- and meso-level interventions considered in this review, which conceptualises the contextual change only in relation to changes in population groups or ways of intervention delivery. This may however also be explained by a lack of explicit consideration of more “natural” adaptations and systems changes during intervention implementation. In any case, this speaks to a lack of in-depth thinking and reporting on context from a broader systems perspective in adaptation practice.

Context has become a central concept in implementation in recent years, and there is growing recognition of the need to better understand context and its interactions with intervention mechanisms in producing outcomes [54]. This is a key feature of an increasingly common complexity perspective which argues for a more holistic and functional view of interventions as embedded within complex systems of interactions [55]. From this perspective, changes to the context to accommodate an intervention are as important as changes to the intervention components to better fit the context [56]. This perspective also supports a functional view of fidelity arguing for standardisation of intervention mechanisms rather than specific components [10]. While there are increasing attempts to operationalise this perspective in intervention development and evaluation [11, 57], its translation into the practice of adaptation is lagging, where the compositional view of interventions (i.e., what they comprise in terms of individual components and which of these are the defining core components) is still predominant. Context is currently used to define different spatial characteristics while ignoring its temporal dimension [2]. Related to this, we found a lack of thinking around programme theories in the examined studies of adaptation, including potential adaptations of the logic models of interventions and the principles underpinning them [58]. This was also the case for the guidance papers, where the compositional rather than functional view of interventions also prevails. In the meantime, lack of in-depth consideration of context and mechanisms of change risks reproducing surface aspects as opposed to underlying mechanisms in the new context.

Overall, the process of adaptation described in the selected cases resonates well with those described in the guidance papers. In the systematic review of guidance on adaptation, we identified 11 unique steps of adaptation: (i) initial assessment, (ii) intervention selection, (iii) intervention exploration, (iv) identification of potential mismatches, (v) intervention model development, (vi) establishment of network, capacity and infrastructure, (vii) undertaking modifications, (viii) pilot (testing), (ix) intervention revision and implementation, (x) evaluation, and (xi) maintenance and evolution. The procedures described in the selected studies cover all of these steps with the exception of maintenance and evolution, which aims to disseminate the adapted interventions and sustain them through further capacity training. There are however gaps in the level of detail and nuance considered across these steps. For example, while studies highlighted intervention selection is a key step, assessment or discussions of the quality of evidence on which intervention selection decisions were made were largely missing.

Both in guidance papers and in studies of adaptations, we found emphasis on involving stakeholders in adaptation. In both cases, a wide range of stakeholders have been described as important to consult at various phases of adaptation. While stakeholder involvement is uniformly viewed as a positive strategy to enhance responsiveness to local needs and facilitate acceptability, we did not find reflections in either guidance papers or studies of adaptation on how potential conflicts may be resolved as a result of involving different groups with different interests (e.g., local stakeholders vs intervention developers) [59]; similarly, there is a lack of discussion as to which stakeholders may need to be prioritised and given specific roles at which phase of adaptation.

Another key gap in the guidance papers on adaptation relates to adaptation re-evaluation. Key questions are whether a full-scale evaluation is required in every case of adaptation or whether a less costly evaluation would be sufficient, and how the decisions on the approaches to re-evaluation should be made. Our review of studies of adaptation shows that pilot and feasibility studies, followed by a full-scale evaluation, are viewed as the ideal approach to evaluating an adapted intervention in practice. However, it may be that where there is only minimal uncertainty, a new large-scale evaluation is not always warranted. Further methodological work is therefore warranted around adaptation re-evaluation to explore whether there might be viable alternative approaches to re-evaluation which would save financial and human resources associated with full-scale evaluations. Aarons and colleagues have put forward conceptual arguments for the adapted interventions to “borrow strength” from the evaluation in the original context, which however require further empirical testing [53]. Another gap in the guidance papers that was highlighted by this scoping review relates to the adaptation of macro-level interventions. As in the guidance papers, macro-level interventions were scarce in the studies considered in this scoping review (*n* = 1). Additional research is therefore needed to examine the adaptation of these broader interventions, as many of the procedures described in current guidance and practice may not be easily applicable to these interventions (e.g., application of CBPR principles to engage policymakers at national-level institutions).

Finally, it is important to highlight the importance of adequate reporting of intervention adaptation. We did not identify any study in this scoping review that referred to guidance for adaptation reporting. In fact, the systematic review of adaptation guidance did not identify any existing guidance providing recommendations on how best to report adaptation. Recently, Stirman and colleagues developed the Framework for Reporting of Adaptations and Modifications to Evidence-based interventions (FRAME) [7]. It provides a systematic approach for considering when and how modifications occur during implementation and allows for reporting of both planned and unplanned adaptations. The use of frameworks such as FRAME will allow for a more transparent understanding of the process of adaptation and how it may influence health and implementation outcomes.

### Strengths and limitations of this scoping review

This scoping review has several strengths. It provides the first systematically drawn database of adapted interventions and thoroughly explores the content of a sample of cases following best practices in reviewing. Informed by the results of a related systematic review of adaptation guidance, it addresses some of the gaps in the scope of current guidance by examining adaptation practices across different intervention levels and topics, contexts, and countries. The review findings, specifically the database of 298 studies of adaptation, can serve as the basis for building a repository of adapted interventions as proposed by Chambers and Norton [60]. This may help to enhance the understanding of the external validity of evidence-informed interventions and provide feedback to practice communities through systematic documentation of the modifications and implementation variations across contexts.

There are a few limitations for this review. While we used a systematic search strategy to identify studies of adaptation, it was primarily designed to retrieve guidance papers on adaptation. The identified studies of adaptation might therefore be more inclined towards reporting a structured process of adaptation (based on the set of terms used in the search strategy, such as guidance, standards, recommendations, and methods). It should be noted, however, that the percentage of studies reporting using adaptation guidance was relatively small (27% of the studies included in the map and 21% of the selected sample), which nevertheless might still overestimate the actual rate of guidance use in practice. Furthermore, as the searches were conducted in English, we might have missed relevant non-English studies. Studies of adaptation of macro-level interventions are highly underrepresented in our review. While there may be different reasons for this, including difficulties with and a lack of formal adaptations of these broader interventions, this can also be reflective of a narrow focus of our search strategy on adaptation and related technical terms (i.e., adaptation, tailoring, transfer, replication). It is possible that adaptation of macro-level interventions is framed and conceptualised differently (e.g., policy change); it is also possible that these adaptations are more likely to be reported outside of academic or in the political sciences literature than in the health sciences literature, which was the main scope of our review. It is also important to note that we had rounds of discussions within the author team regarding the coding of the levels of intervention. Many interventions that we assigned to the meso-level could also be viewed as micro-level, depending on the perspective taken. Finally, as is the case for all types of reviews, the findings of our review are limited by the reporting of the included studies. We will however aim to address the possible lack of reporting of additional aspects of adaptation through qualitative interviews with stakeholders in subsequent stages of the ADAPT study.

## Conclusions

This scoping review provides a map of adaptation studies across multiple topics and types of interventions. It offers a database of 298 adapted interventions describing the contexts, methods, and interventions considered for adaptation, and in-depth narrative accounts of the rationale, types, and procedures for adapting interventions to new contexts and practice-based approaches to re-evaluation. It addresses some of the gaps identified in the previous systematic review of adaptation guidance, such as looking into adaptation across different countries [5], but other gaps remain, such as adaptation of macro-level interventions, consideration of programme theories, mechanisms and contexts (i.e., a functional view of interventions), nuances around stakeholder involvement, and guidance for decision-making around appropriate evaluation of the adapted interventions.

## Supplementary Information

**Additional file 1.** Preferred Reporting Items for Systematic Reviews and Meta-Analyses extension for Scoping Reviews (PRISMA-ScR) Checklist.

**Additional file 2.** Search strategy.

**Additional file 3.** Extraction form.

**Additional file 4.** Map of adaptation studies.

## Data Availability

Further data and materials are included in the additional files.

## Abbreviations

ADAPT-ITT: Assessment–Decision–Administration–Production–Topical Experts–Integration–Training–Testing
CBPR: Community-based participatory research
EBI: Evidence-based intervention
HIV: Human immunodeficiency virus (HIV)
PRISMA-ScR: Preferred Reporting Items for Systematic Reviews and Meta-Analysis extension for Scoping Reviews
sIMB-CIM: Situated-Information Motivation Behavioural Skills Model of Care Initiation and Maintenance
STD: Sexually transmitted disease
STI: Sexually transmitted infection

## Footnotes

1 Micro-level interventions were defined as those intervening with individuals and their immediate social network and relationships, such as the family. Meso-level interventions were defined as those focusing on intervening with medium-level population groups, such as neighbourhoods and schools. Here, intervention mechanisms often focused on institutional or cultural change. Macro-level interventions were those that intervened with overarching social systems operating at the national or global level, such as through regulations, taxation, other government policies, or mass media interventions.
